# Statistical Topology—Distribution and Density Correlations of Winding Numbers in Chiral Systems

**DOI:** 10.3390/e25020383

**Published:** 2023-02-20

**Authors:** Thomas Guhr

**Affiliations:** Fakultät für Physik, Universtät Duisburg–Essen, 47048 Duisburg, Germany; thomas.guhr@uni-due.de

**Keywords:** statistical topology, random matrices, chirality, winding numbers

## Abstract

Statistical Topology emerged as topological aspects continue to gain importance in many areas of physics. It is most desirable to study topological invariants and their statistics in schematic models that facilitate the identification of universalities. Here, the statistics of winding numbers and of winding number densities are addressed. An introduction is given for readers with little background knowledge. Results that my collaborators and I obtained in two recent works on proper random matrix models for the chiral unitary and symplectic cases are reviewed, avoiding a technically detailed discussion. There is a special focus on the mapping of topological problems to spectral ones as well as on the first glimpse of universality.

## 1. Introductory Remarks

Statistical Topology aims at combining, in a generalizing form, topological questions appearing in physics with the powerful concepts of Random Matrix Theory (RMT) which is capable of describing spectral statistics in a huge number of systems, stemming from different areas of physics and beyond. The focus in this work is exclusively on winding numbers and associated statistical quantities studied in the framework of a random matrix model; other topological invariants, such as the Chern numbers, which are also of considerable interest, are not discussed. The long-term aim is to study the emergence of universalities whose identification and usage is always, in all branches of statistical physics, the most rewarding enterprise. I have two goals. First, I want to present an introduction to Statistical Topology, restricted to statistical problems which are related to winding numbers, for readers without a pertinent background. Neither physics expert jargon, nor heavy mathematics and mathematical physics terminology are used. Second, I want to review and summarize results that my collaborators and I obtained in two recent studies [1,2]. We calculated for a chiral unitary random matrix model correlators of winding number densities and the winding number distribution. We also computed generators for these correlators in a chiral unitary and a chiral symplectic random matrix model. Furthermore, we made first steps towards finding universalities.

The paper is organized as follows: In Section 2, the salient features of winding numbers and chiral symmetry are presented. In Section 3, a schematic model with the necessary mathematical setup is formulated. Results are reviewed in Section 4, discussion and conclusions are given in Section 5.

## 2. Winding Numbers and Chirality

After briefly revisiting the occurrence of winding numbers in complex analysis in Section 2.1, the Kitaev chain is discussed in Section 2.2 and the statistical ansatz is motivated in Section 2.3. The research is placed in the framework of Quantum Chromodynamics (QCD) and Condensed Matter Physics in Section 2.4, summarizing the corresponding remarks in Refs. [1,2].

### 2.1. A Simple Topological Invariant in Complex Analysis

**Figure 1:**
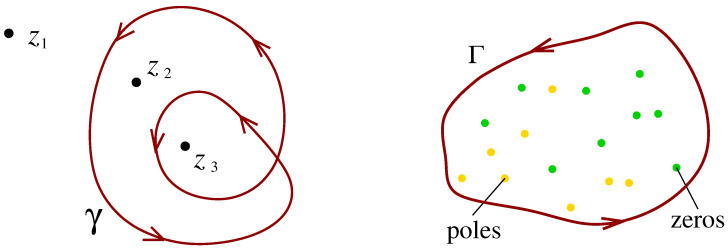
**Left**: Three points $z_{i},\;i=1,2,3$ in the complex plane C and a closed contour γ. **Right**: A closed contour Γ encircling zeros and poles of a meromorphic function f(z).

The winding number is a topological concept encountered in complex analysis. Before discussing applications in physics, we briefly sketch the mathematical background. The winding number $W=W(z_{i})$ counts how many times a point $z_{i}$ in the complex plane C is encircled by a closed contour γ, where counterclockwise or clockwise give a positive or a negative contribution, respectively. An example is shown in Figure 1, we have $W(z_{1})=0$, $W(z_{2})=1$ and $W(z_{3})=2$. Obviously, the winding number $W(z_{i})$ is a topological constant or, in physics terminology, a quantum number. It is invariant under all deformations of γ that do not cross the point $z_{i}$ in question. In particular, the winding number is always a positive or negative integer, W∈Z. It may be written as the contour integral

(1) $$W\left(z_{i}\right)=\frac{1}{2\pi i}\oint_{\gamma }\frac{d\zeta }{\zeta -z_{i}}\;.$$

One easily establishes the link to Cauchy’s argument principle: Consider a meromorphic function f(z) and a closed contour Γ, encircling some zeros and poles of f(z) in the complex plane C as shown in the example in Figure 1. The integral along this contour Γ over the logarithmic derivative of f(z) yields the difference of the number $N_{Z}$ of zeros and the number $N_{P}$ of poles, hence

(2) $$\frac{1}{2\pi i}\oint_{\Gamma }\frac{f^{\prime }\left(z\right)}{f(z)}dz=N_{Z}-N_{P}\;.$$

The close relation to the winding number is found by making the change of variable ζ=f(z) and in accordance with the contour, Γ→f(Γ),

(3) $$N_{Z}-N_{P}=\frac{1}{2\pi i}\oint_{\Gamma }\frac{f^{\prime }\left(z\right)}{f(z)}dz=\frac{1}{2\pi i}\oint_{f(\Gamma )}\frac{d\zeta }{\zeta }=W\left(0\right).$$

We conclude that $N_{Z}-N_{P}$ is the winding number W(0) of the closed contour f(Γ) around the origin z=0. As, from now on, all winding numbers will refer to the origin, we drop the argument and simply write *W*.

### 2.2. Kitaev Chain and Winding Numbers

**Figure 2:**
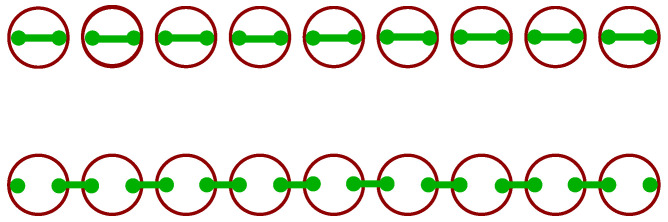
Kitaev chain, electrons as larger open circles (red), Majorana fermions as small dots (green) with the pairing indicated by connecting lines (green). Top: All Majorana fermions are paired, normal or trivial superconducting phase. Bottom: Unpaired Majorana fermions at the ends of the chain, topological superconducting phase.

To illustrate the occurrence of topological invariants in physics, we look at the Kitaev chain [3,4] as a prominent example. It consists of spinless electrons with next-neighbor hopping and superconductive pairing. The Hamiltonian reads, in a slightly simplified form sufficient for the present discussion,

(4) $$\hat{H}=\sum_{n}\left(t\left({\hat{c}}_{n}^{†}{\hat{c}}_{n+1}+{\hat{c}}_{n+1}^{†}{\hat{c}}_{n}\right)+\mu {\hat{c}}_{n}^{†}{\hat{c}}_{n}+\frac{\Delta }{2}\left({\hat{c}}_{n+1}^{†}{\hat{c}}_{n}^{†}+{\hat{c}}_{n}{\hat{c}}_{n+1}\right)\right)\;,$$

where ${\hat{c}}_{n}$ and ${\hat{c}}_{n}^{†}$ are annihilation and creation operators, respectively, at position *n* on the chain. Moreover, μ and Δ are chemical and pairing potentials and *t* is the hopping strength. The Hamiltonian may be reformulated in terms of Majorana fermions whose number is twice that of the electrons. Remarkably, depending on the parameters, there are two possibilities, as schematically depicted in Figure 2. Either all Majorana fermions are paired or, at the ends of the chain, two of them are unpaired [5]. In the former case, the chain is in a normal or trivial superconducting phase, in the latter, in a topological one. This aspect deserves further discussion.

In Fourier space, the Kitaev chain corresponds to the Bloch–Bogolyubov–de Gennes Hamiltonian matrix H(k). It is a crucial that this 2×2 matrix satisfies chiral symmetry,

(5) $$\begin{array}{c} \left\{H\left(k\right),C\right\}=0\;\mathrm{with}\;C=\left[\begin{array}{cc} 1 & 0\\ 0 & -1 \end{array}\right]\;. \end{array}$$

The matrix C is the chiral operator in its proper basis and {,} is the anticommutator. It is then possible to write the Hamiltonian matrix in the form

(6) $$\begin{array}{c} H\left(k\right)=\vec{d}\left(k\right)\cdot \vec{\sigma }\;\mathrm{with}\;\vec{d}\left(k\right)=\left(0,\Delta \sin k,\mu +2t\cos k\right)\;. \end{array}$$

Hence, using the three-component vector $\vec{\sigma }$ of the 2×2 Pauli matrices, H(k) is found to be a scalar product with all physics encoded in the vector $\vec{d}\left(k\right)$ that depends on the wave number *k* and the three parameters μ, Δ and *t*. Importantly, the first component is zero, $d_{x}=0$. This restriction to effectively only two dimensions can be shown to be a consequence of chiral symmetry (5).

**Figure 3:**
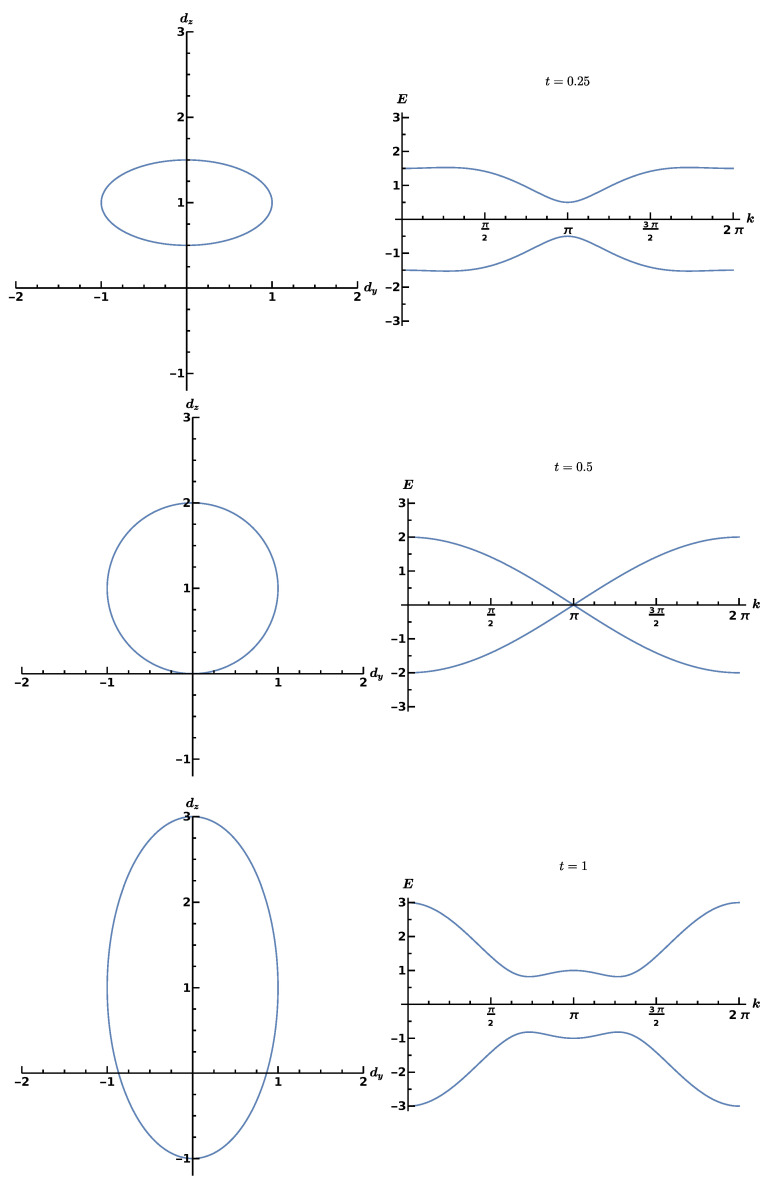
Ellipses described by $\vec{d}\left(k\right)$ (**left**) and corresponding dispersion relations E(k) (**right**). **Top**: t=0.25, normal superconducting phase, W=0. **Center**: t=0.5, phase transition point. **Bottom**: t=1, topological superconducting phase, W=1. Courtesy of Nico Hahn.

To see how topology enters, we notice that the vector $\vec{d}\left(k\right)$ describes an ellipse with parameter *k* on the curve, μ determines the position of its center, Δ and *t* determine its shape. In Figure 3, we depict $\vec{d}\left(k\right)$ for fixed values of μ=1, Δ=1 and three different values t=0.25,0.5,1 with the corresponding energy dispersion relation E(k). If the origin of the (y,z) plane is included in the closed contour that the ellipse describes, its winding number is one, W=1. If not, the winding number is zero, W=0. These are two topologically separated scenarios, reflecting the distinctly different role of the Majorana fermions in the top and bottom parts of Figure 2. For W=0, the superconducting phase is the normal or trivial one, while it is topological for W=1. A special situation occurs if the ellipse just touches the *x* axis, the band gap disappears, marking the phase transition point.

### 2.3. Chirality, Random Winding Numbers and Modelling Aspects

When studying such topological invariants in statistical physics, the closed contour might be a random quantity, for example, generated by a proper ensemble of Hamiltonians. In the case of the Kitaev chain, this ensemble may be realized by choosing the parameters μ, Δ and *t* from probability distributions. Hence, the contour can be different for a particular choice, i.e., it becomes random, and the winding number *W* will be random as well. In general, the dynamics of a system under consideration, described by the Hamiltonian, and the distributions of its parameters will determine the distribution P(W). Are there universalities when comparing different systems? If yes, in which quantities do these universalities manifest? In the distributions P(W) on their original scales or on some scales which make these systems comparable? These are the guiding questions for our research. Universalities are best identified in random schematic systems that only contain the most basic ingredients needed for the relevant physics, in the present case for the occurence of winding numbers. Random Matrix Theory (RMT) [6,7] is known to be a powerful concept in this spirit when studying universalities in spectral correlations as well as in the correlations of parametric level motion [8,9]. The chiral symmetry (5) and, thus, the restriction to two dimensions are essential for the interpretation of the two superconducting phases in the Kitaev chain in terms of the winding number. Hence, when setting up a schematic random matrix model, we need to employ chirality.

### 2.4. Connections to Quantum Chromodynamics and Condensed Matter Physics

In Quantum Chromodynamics, the chiral symmetry of the Dirac operator is broken spontaneously as well as explicitly by the quark masses. The chiral condensate is the order parameter of the phase transition that occurs at a high temperature and that restores chiral symmetry, which is related to the confinement–deconfinement transition. To investigate statistical properties of lattice gauge calculations, chiral RMT [10,11,12,13,14,15,16,17] is remarkably successful. As in the original RMT, presence or absence of time-reversal invariance combined with spin-rotation symmetries results in three classes of chiral random matrices: orthogonal, unitary and symplectic. It was then shown that altogether ten RMT symmetry classes [18,19,20,21,22] exist, referred to as the tenfold way. The three original and the three chiral ones comprise six of these ten classes, the remaining four emerge when particle-hole symmetry is also considered, see Refs. [23,24]. In condensed matter physics, chiral symmetry is realized by sublattice symmetry (see early work in Ref. [25]) or as a combination of time reversal and particle-hole symmetry [24].

In the terminology of condensed matter physics, the winding number comes in as characterization of translationally invariant one-dimensional chiral systems that are gapped at the centre of the spectrum. The winding number is the integer topological index with respect to the bundle of negative-energy bands. A non-zero winding number *W* indicates the topologically non-trivial situation with |W| modes at each boundary [26,27,28,29]. The winding number differs for different realization of the disorder, i.e., it becomes random. Our research on the winding number was inspired by studies of systems with energy bands in two dimensions, allowing for a topological classification by the (first) Chern number. A random matrix model [30,31] revealed a Gaussian distribution of Chern numbers with a universal covariance.

Another intriguing direction might be the application of Statistical Topology to classical wave phenomena such as microwaves or acoustics and, furthermore, to photonics where topological issues are already in focus [32].

## 3. Formulation of the Problem and Mathematical Setup

After introducing chiral random matrix ensembles with a parameter dependence in Section 3.1, the statistical quantities of interest are defined in Section 3.2. In Section 3.3, a crucial step for all of our mathematical investigations is presented, namely, the mapping of the topological problem addressed to a spectral one which greatly facilitates the computations.

### 3.1. Chiral Random Matrix Ensembles with Parametric Dependence

We derived results [1,2] for the chiral unitary and the chiral symplectic symmetry classes labeled AIII and CII, respectively, see Ref. [18]. The latter case is mathematically much more demanding than the former, but not as involved as the orthogonal case, labeled BDI. Only very recently have we been able to solve it, this will be published elsewhere. The cases BDI and CII describe time-reversal invariant systems, while this invariance does not exist in the case AIII. We refer to the matrices as Hamiltonians *H*, as most of the present application of winding numbers seem to stem from Condensed Matter Physics. The matrices *H* are complex Hermitean or quaternion real, i.e., self-adjoint, with even dimension βN×βN where we employ the Dyson indices β=2 and β=4 for AIII and CII. Chiral symmetry manifests in the relation

(7) {C,H}=0

where in the chiral basis

(8) $$C=\left[\begin{array}{cc} 1\;1_{\beta N/2} & 0\\ 0 & -1\;1_{\beta N/2} \end{array}\right]\;.$$

The Hamiltonians thus take the block off-diagonal form

(9) $$H=\left[\begin{array}{cc} 0 & K\\ K^{†} & 0 \end{array}\right]\;,$$

where the βN/2×βN/2 matrices *K* have no further symmetries. We draw the matrices *H* from the chiral Gaussian Unitary, respectively, Symplectic Ensembles (chGUE, chGSE). To study questions of topology, we give these random matrices a parametric dependence K=K(p) and thus, H=H(p), where the real variable *p* lies on the unit circle. The winding number corresponding to these Hamiltonians is then [33,34]

(10) $$W=\frac{1}{2\pi i}\int_{0}^{2\pi }w\left(p\right)\;dp\;,$$

with the winding number density

(11) $$w\left(p\right)=\frac{d}{dp}\ln\det K\left(p\right)=\frac{1}{\det K(p)}\frac{d}{dp}\det K\left(p\right)\;.$$

Cauchy’s argument principle applies to the integral (10), provided detK is a non-zero analytic function of *p*, see Section 2.1 and particularly, Equation (3).

To produce explicit results, we choose a particular realization of the parameter dependence. With two smooth and 2π periodic scalar functions a(p) and b(p), we set

(12) $$K\left(p\right)=a\left(p\right)K_{1}+b\left(p\right)K_{2}\;,$$

where the matrices $K_{1}$ and $K_{2}$ have dimensions βN/2×βN/2. The associated Hamiltonians

(13) $$H\left(p\right)=a\left(p\right)H_{1}+b\left(p\right)H_{2}\;\mathrm{with}\;H_{m}=\left[\begin{array}{cc} 0 & K_{m}\\ K_{m}^{†} & 0 \end{array}\right]\;,\;m=1,2\;,$$

define parametric combinations of either two chGUE’s or two chGSE’s. Averages over these combined ensembles have to be performed. It is convenient to introduce the vector

(14) $$v\left(p\right)=\left(a\left(p\right),b\left(p\right)\right)\;\in C^{2}\;.$$

Time-reversal invariance imposes the condition $v^{*}\left(p\right)=v\left(-p\right)$ in the chiral symplectic case CII.

### 3.2. Statistical Quantities Considered

Considering *k* different points $p_{i},\;i=1,\ldots ,k$, on the unit circle, we are interested in the *k*-point correlators of winding number densities

(15) $$C_{k}^{\left(\beta ,N\right)}\left(p_{1},\ldots ,p_{k}\right)=\langle w\left(p_{1}\right)\cdots w\left(p_{k}\right)\rangle$$

The precise meaning of the angular brackets indicating the ensemble average will be given later on. In the chiral unitary case AIII, we computed these correlators directly [1], see Section 4.1. As, first, this approach becomes forbiddingly complicated in the chiral symplectic case CII, and, second, results in cumbersome expressions for larger *k*, we calculated the generators

(16) $$Z_{k|l}^{\left(\beta ,N\right)}\left(q,p\right)=\langle \frac{\prod_{j=1}^{l}\det K\left(p_{j}\right)}{\prod_{j=1}^{k}\det K\left(q_{j}\right)}\rangle$$

for two sets of variables $p_{1},\ldots ,p_{l}$ and $q_{1},\ldots ,q_{k}$ in Ref. [2], see Section 4.4. Only the case k=l is needed, but the more general definition (16) for *k* and *l* being different has technical advantages. We notice that *k* and *l* are the numbers of determinants in denominator and numerator, respectively. The *k*-fold derivative

(17) $$C_{k}^{\left(\beta ,N\right)}\left(p_{1},\ldots ,p_{k}\right)=\frac{\partial^{k}}{\prod_{j=1}^{k}\partial p_{j}}Z_{k|k}^{\left(\beta ,N\right)}\left(q,p\right){|}_{q=p}$$

of the generator (16) for k=l at q=p yields the correlator (15). Anticipating the later discussion, we emphasize that the generators for both Dyson indices β=2,4 will exhibit a remarkably clear structure [2] which is an important reason to address them here. It is worth mentioning that the correlators (15) and the generators (16) are very different from those for the parametric level motion considered in Refs. [8,9].

Furthermore, we also computed the distribution of winding numbers P(W) in the chiral unitary case AIII [1], see Section 4.2.

### 3.3. Mapping a Topological to a Spectral Problem

At first sight, the computation of the correlators (15) and the generators (16) appears as a formidable task, requiring the development of completely new techniques in RMT. Luckily, one can establish a link between the topological problem set up above and spectral problems in RMT for which a wealth of literature exists. This amounts to a tremendous simplification, even though the calculations to be performed are still involved and quite demanding, particularly in the chiral symplectic case. The key observation is that a combination of the two matrices $K_{1}$ and $K_{2}$ in Equation (12) encodes all the statistical information needed. Pulling out $K_{1}$, say, one has

(18) $$K\left(p\right)=a\left(p\right)K_{1}+b\left(p\right)K_{2}=b\left(p\right)K_{1}\left(\kappa \left(p\right)1\;1_{\beta N/2}+K_{1}^{-1}K_{2}\right)$$

with the ratio

(19) $$\kappa \left(p\right)=\frac{a(p)}{b(p)}\;.$$

Since the winding number density (11) is the derivative of the logarithm

(20) $$\ln\det K\left(p\right)=\ln\det K_{1}+\beta N\ln b\left(p\right)+\ln\det\left(\kappa \left(p\right)+K_{1}^{-1}K_{2}\right)\;,$$

the first term $\ln\det K_{1}$ does not contribute and, remarkably, only the combination $Y=K_{1}^{-1}K_{2}$ is relevant. Using Equation (18), the generators acquire the form

(21) $$Z_{k|k}^{\left(\beta ,N\right)}\left(q,p\right)={\left(\prod_{j=1}^{k}\frac{b(p_{j})}{b(q_{j})}\right)}^{\beta N}\langle \prod_{j=1}^{k}\frac{\det(\kappa \left(p_{j}\right)1\;1_{\beta N/2}+Y)}{\det(\kappa \left(q_{j}\right)1\;1_{\beta N/2}+Y)}\rangle \;,$$

which as well only contains the matrix *Y*.

The task to be solved is the derivation of the probability density for the random matrices $Y=K_{1}^{-1}K_{2}$ from the independent Gaussian distributions for the random matrices $K_{1}$ and $K_{2}$. Once again, luckily, the results are known as spherical [35,36] ensembles and their probability densities read explicitly

(22) $${\tilde{G}}^{\left(\beta \right)}\left(Y\right)=\frac{1}{\pi^{\beta N^{2}/2}}\prod_{j=1}^{N}\frac{\left(\beta (N+j)/2-1\right)!}{\left(\beta j/2-1\right)!}\;\frac{1}{\det^{2N}\left(1\;1_{\beta N/2}+YY^{†}\right)}\;.$$

These ensembles are referred to as complex spherical and quaternion spherical for β=2,4. In the complex case, the probability density (22) can be reduced to a joint probability density of the *N* complex eigenvalues $z=\mathrm{diag}\;(z_{1},\ldots ,z_{N})$ of *Y* and reads

(23) $$G^{\left(2\right)}\left(z\right)=\frac{1}{c^{\left(2\right)}}{\left|\Delta_{N}\left(z\right)\right|}^{2}\prod_{j=1}^{N}\frac{1}{\left(1+\right|z_{j}{{|}^{2})}^{N+1}}$$

with the the Vandermonde determinant

(24) $$\Delta_{N}\left(z\right)=\prod_{j<l}\left(z_{j}-z_{l}\right)\;.$$

In the quaternion case, however, each eigenvalue $z_{j}$ of *Y* has a complex conjugate $z_{j}^{*}$, which is also an eigenvalue. The corresponding joint probability density of the eigenvalues $z=\mathrm{diag}\;(z_{1},z_{1}^{*},z_{2},z_{2}^{*},\ldots ,z_{N},z_{N}^{*})$ is given by

(25) $$G^{\left(4\right)}\left(z\right)=\frac{1}{c^{\left(4\right)}}\Delta_{2N}\left(z\right)\prod_{j=1}^{N}\frac{z_{j}-z_{j}^{*}}{\left(1+\right|z_{j}{{|}^{2})}^{2N+2}}\;.$$

The normalization constants are

(26) $$c^{\left(\beta \right)}={\left(\frac{\beta \pi }{2}\right)}^{N}N!\prod_{j=1}^{N}B\left(\frac{\beta j}{2},\frac{\beta (N+1-j)}{2}\right)\;,$$

where B(x,y) is Euler’s Beta function. The question whether the integrals to be calculated are well-defined for β=4 arises, but the answer is affirmative [2]. Hence, the ensemble average over a function f(z) to be performed amounts to carrying out the integral

(27) $$\langle f\left(z\right)\rangle =\int_{C}d\left[z_{1}\right]\cdots \int_{C}d\left[z_{N}\right]\;G^{\left(\beta \right)}\left(z\right)\;f\left(z\right)$$

over all complex eigenvalues. Hence, by reducing the two chiral ensembles to a single spherical one for either β, all information of the topological problem is contained in the determinants $\det(\kappa \left(p\right)1\;1_{\beta N/2}+Y)$ or their derivatives. Most advantageously, this is equivalent to a spectral problem where *Y* and κ(p) formally play the roles of a (complex or quaternion) “Hamiltonian” and of the corresponding “energy”, respectively.

## 4. Results

The correlators for the unitary case are addressed in Section 4.1, the distribution is given in in Section 4.2. Aspects of universality are discussed in Section 4.3. The generators in the chiral unitary and symplectic cases are presented in Section 4.4.

### 4.1. Winding Number Correlators in the Chiral Unitary Case

In Ref. [1], we calculated the winding number correlators $C_{k}^{\left(2,N\right)}\left(p_{1},\ldots ,p_{k}\right)$ as defined in Equation (15) in the unitary case directly. We chose

(28) a(p)=cospandb(p)=sinp.

Using Equations (11) and (20) as well as the complex eigenvalues of *Y*, one has

(29) $$w\left(p\right)=N\cot p+y\left(p\right)\;\mathrm{with}\;y\left(p\right)=-\frac{1}{\sin^{2}p}\sum_{n=1}^{N}\frac{1}{\cot p+z_{n}}\;.$$

Only the *k*-fold products of y(p) have to be ensemble averaged with the joint probability density (23), the presence of the inconvenient term Ncotp implies that the correlator $C_{k}^{\left(2,N\right)}\left(p_{1},\ldots ,p_{k}\right)$ of the *k* winding number densities $w(p_{j})$ becomes a combinatorial sum of the $y(p_{j})$ correlators. Furthermore, the latter themselves turn out to be rather involved combinatorial expressions. Eventually, $C_{k}^{\left(2,N\right)}\left(p_{1},\ldots ,p_{k}\right)$ is found to be a combinatorial sum of determinants with the entries

(30) $$L_{nml}\left(q_{l}\right)=\frac{{\left(-1\right)}^{m-n}\pi }{q_{l}^{m-n+1}}B\left(m,N-m+1\right)\left\{\begin{array}{cc} u_{m}\left(N,q_{l}^{2}\right)\; & m\geq n\\ -v_{m}\left(N,q_{l}^{2}\right)\; & m<n \end{array}\right.$$

with the properly normalized incomplete Beta functions

(31) $$\begin{array}{ccc} u_{m}\left(N,q_{l}^{2}\right) & = & \frac{2}{B(m,N-m+1)}\int_{0}^{q_{l}}d\rho \frac{\rho^{2m-1}}{{\left(1+\rho^{2}\right)}^{N+1}}\\ v_{m}\left(N,q_{l}^{2}\right) & = & \frac{2}{B(m,N-m+1)}\int_{q_{l}}^{\infty }d\rho \frac{\rho^{2m-1}}{{\left(1+\rho^{2}\right)}^{N+1}} \end{array}$$

that satisfy $u_{m}\left(N,q_{l}^{2}\right)+v_{m}\left(N,q_{l}^{2}\right)=1$. Even though B(m,N−m+1) drops out in the $L_{nml}\left(q_{l}\right)$, this normalization has advantages, see Ref. [1]. The first two correlators read

(32) $$\begin{array}{ccc} C_{1}^{\left(2,N\right)}\left(p_{1}\right) & = & 0\\ C_{2}^{\left(2,N\right)}\left(p_{1},p_{2}\right) & = & -\frac{1-\cos^{2N}\left(p_{1}-p_{2}\right)}{1-\cos^{2}\left(p_{1}-p_{2}\right)}\;. \end{array}$$

The at-first-sight surprising vanishing of the averaged winding number density is actually quite natural, as the winding number *W* must have a symmetric distribution with vanishing first moment. The integral of $C_{1}^{\left(2,N\right)}\left(p_{1}\right)$ over $p_{1}$ is this first moment.

### 4.2. Winding Number Distribution

In Ref. [1], we also computed the winding number distribution P(W) in the unitary case for the choice (28). Using Cauchy’s argument principle, we derive the discrete probability distribution

(33) P(W)=rW+N2N(W+N)/2

on the integers *W* between −N and +N for arbitrary, finite matrix dimension *N*. Here, r(m) is the probability that *m* eigenvalues are inside the unit circle and the remaining ones outside which may be written as

(34) $$r\left(m\right)=\int_{|z_{1}|<1}d\left[z_{1}\right]\cdots \int_{|z_{m}|<1}d\left[z_{m}\right]\int_{|z_{m+1}|>1}d\left[z_{m+1}\right]\cdots \int_{|z_{N}|>1}d\left[z_{N}\right]\;G^{\left(2\right)}\left(z\right)\;.$$

Calculating the integrals yields

(35) $$r\left(m\right)=\frac{1}{N!}\sum_{\omega \in S_{N}}\left(\prod_{i=1}^{m}u_{\omega (i)}\left(N,1\right)\right)\left(\prod_{i=m+1}^{N}v_{\omega (i)}\left(N,1\right)\right),$$

in terms of the functions (31). The combinatorial factor in Formula (33) takes into account the permutation invariance of the eigenvalues inside, respectively, outside, the unit circle. The sum runs over all permutations, $S_{N}$ is the permutation group.

### 4.3. Aspects of Universality

The quest for universality is twofold, first, there is the question of whether the same statistical effects, distributions or scalings, etc, can be identified in empirical or experimental data of different physical systems. Second, there is the theoretical and mathematical side concerned with often schematic models and their ability to describe or even predict the results from data analysis. In the case of spectral correlations, universal statistics is found on the local scale of the mean level spacing, i.e., universalities are revealed after a rescaling of the energies, referred to as unfolding. The unfolded correlators of, on the one hand, RMT for infinite level number and of, on the other hand, numerous physical systems of very different nature with large number of levels coincide, see the discussion in Refs. [6,7]. The theoretical and mathematical challenge is non-trivial as it amounts to showing that a most general class of probability densities for the random matrices yields after unfolding the same statistical quantities. Put differently, it suffices to consider Gaussians because the resulting statistics is, always after unfolding, universal.

In the case of statistical topology, universality is of equally high importance, but it appears to be considerably more complicated. Already, on the theoretical and mathematical side, there are several natural questions to be posed: First, is there an unfolding scale comparable to the local mean level spacing and how is it related to the scale of the level velocity as in the parametric correlations [8,9,37]? Second, which probability densities for the random matrices yield in the model set up in Section 3.1 the same statistics? Third, what are the conditions on the functions a(p) and b(p) or, more precisely, the combined conditions on these functions and the probability densities that yield in the model universal statistics? Fourth, is it possible to find universal statistics for models more general than the one in Section 3.1?

In Ref. [1], we started addressing these issues in the unitary case for the choice (28). Of course, this limits our discussion, a future fully fledged investigation ought to also consider the impact of different choices for these functions. Guided by unfolding in spectral statistics, we rescaled the arguments $p_{i}$ in the correlation functions $C_{k}^{\left(2,N\right)}\left(p_{1},\ldots ,p_{k}\right)$ according to

(36) $$\psi_{i}=N^{\alpha }p_{i}\;.$$

The power α should be positive because we want to zoom into the parametric dependence in the limit N→∞. Consider the two-point function (32) and the limit

(37) $$\lim_{N\to \infty }C_{2}^{\left(2,N\right)}\left(\frac{\psi_{1}}{N^{\alpha }},\frac{\psi_{2}}{N^{\alpha }}\right)\frac{d\psi_{1}}{N^{\alpha }}\frac{d\psi_{2}}{N^{\alpha }}=f_{2}^{\left(\alpha \right)}\left(\psi_{1},\psi_{2}\right)d\psi_{1}d\psi_{2}$$

defining the function $f_{2}^{\left(\alpha \right)}$, if existing. A straightforward calculation yields

(38) $$f_{2}^{\left(\alpha \right)}\left(\psi_{1},\psi_{2}\right)=\left\{\begin{array}{cc} -\frac{1}{{\left(\psi_{1}-\psi_{2}\right)}^{2}}\; & \alpha <\frac{1}{2}\\ -\frac{1-\exp(-{\left(\psi_{1}-\psi_{2}\right)}^{2})}{{\left(\psi_{1}-\psi_{2}\right)}^{2}}\; & \alpha =\frac{1}{2}\\ 0\; & \alpha >\frac{1}{2} \end{array}\right.\;.$$

**Figure 4:**
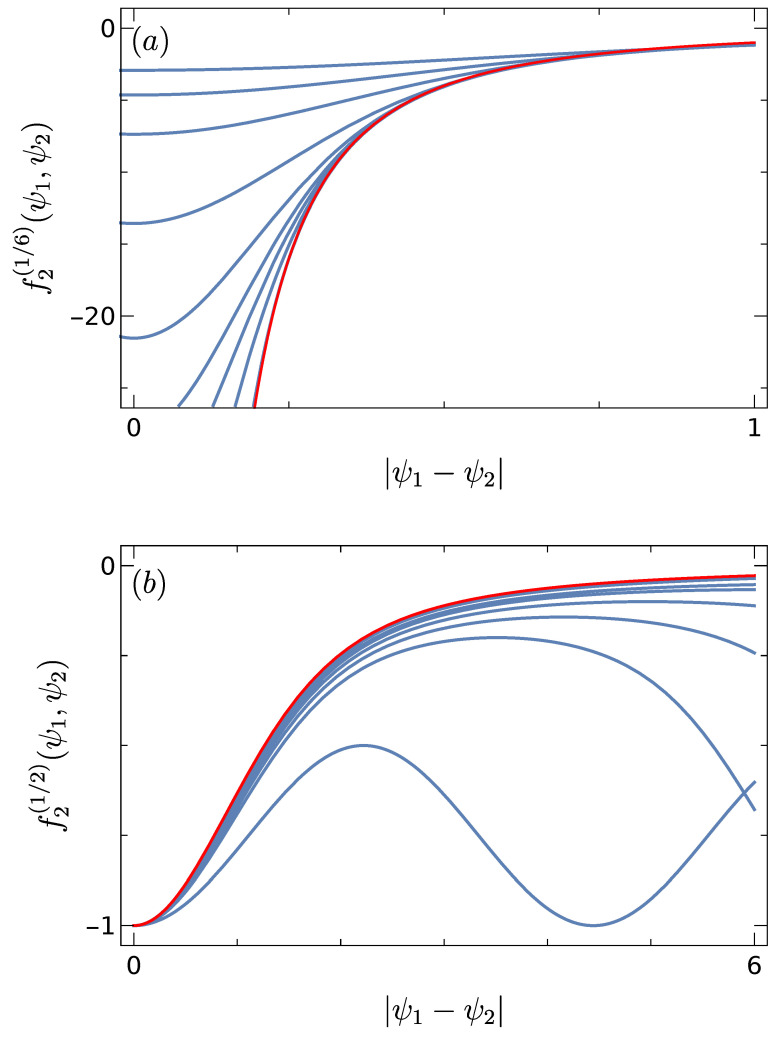
Unfolded two-point function after the rescaling (36) for different values of *N* (blue). In (**a**), we used N=5,10,20,50,100,150,200,300,1000 and α=1/6, in (**b**) N=2,5,7,10,15,20,50,100 and α=1/2. For comparison, the limit (37) is presented (red). Taken from Ref. [1].

We notice $C_{2}^{\left(2,N\right)}\left(p_{1},p_{1}\right)=-1$, see Equation (32), implying that $\psi_{1}\neq \psi_{2}$ when taking the limit for arbitrary α. The result (38) reveals different regimes, the one for α=1/2 involves the same scale as in Refs. [8,9]. Figure 4 shows results for two values of α and various values of *N*, the unfolded two-point function approaches the limit (38) when *N* increases. We conjectured that the function $f_{2}^{\left(\alpha \right)}\left(\psi_{1},\psi_{2}\right)$ is universal [1].

In Ref. [1], we also showed that the winding number distribution (33) becomes Gaussian for large *N*. More precisely, its second moment behaves like $\langle W^{2}\rangle ∼\sqrt{N}$, suggesting an unfolding of the form $W/N^{1/4}$, i.e., different from the rescaling above. It then follows that P(W) approaches a Gaussian with variance $2\sqrt{N/\pi }$ for large *N*.

### 4.4. Generators in the Chiral Unitary and Symplectic Cases

We computed the generators (16), respectively, (21) exactly for β=2 and β=4 in Ref. [2]. To this end, we used the method proposed some years ago in Refs. [38,39]. It identifies and employs, in ordinary space, supersymmetric structures deeply rooted in the ensemble averages. As there is no mapping performed of the ensemble averages to superspace, the method is often referred to, jokingly, but not deceptively, as “supersymmetry without supersymmetry”. In the chiral unitary case β=2, we found a ratio of two determinants,

(39) $$Z_{k|k}^{\left(2,N\right)}\left(q,p\right)=\frac{\det{\left[\frac{1}{v^{T}\left(q_{m}\right)\sigma_{2}v\left(p_{n}\right)}{\left(\frac{v^{†}\left(q_{m}\right)v\left(p_{n}\right)}{v^{†}\left(q_{m}\right)v\left(q_{m}\right)}\right)}^{N}\right]}_{1\leq m,n\leq k}}{\det{\left[\frac{1}{v^{T}\left(q_{m}\right)\sigma_{2}v\left(p_{n}\right)}\right]}_{1\leq m,n\leq k}}\;,$$

where $\sigma_{2}$ is the second 2×2 Pauli matrix and $v(p_{n})$ is the vector defined in Equation (14). In the chiral symplectic case β=4, we arrived at a ratio of a Pfaffian and a determinant,

(40) $$Z_{k|k}^{\left(4,N\right)}\left(q,p\right)=\frac{\mathrm{Pf}{\left[\begin{array}{cc} {\hat{K}}_{1}\left(p_{m},p_{n}\right) & {\hat{K}}_{2}\left(p_{m},q_{n}\right)\\ -{\hat{K}}_{2}\left(p_{n},q_{m}\right) & {\hat{K}}_{3}\left(q_{m},q_{n}\right) \end{array}\right]}_{1\leq m,n\leq k}}{\det{\left[\frac{1}{iv^{T}\left(q_{m}\right)\sigma_{2}v\left(p_{n}\right)}\right]}_{1\leq m,n\leq k}}\;.$$

The three kernel functions ${\hat{K}}_{l}\left(p_{m},p_{n}\right)\;,l=1,2,3$ are quite complicated and can be found explicitly in Ref. [2]. Considering the complexity of the problem and of its mathematical structure, these are remarkably compact results, even in the chiral symplectic case. This compactness is the reason why we present these results here. Their form is intimately connected with the mapping of the topological to a spectral problem discussed in Section 3.3 because such determinant and Pfaffian expressions are ubiquitous for the generators in spectral statistics. Importantly, this carries over, at least for the model considered, to the generators for the correlators of winding number densities.

## 5. Discussion and Conclusions

Statistical Topology is an emerging branch in statistical physics, with connections to various branches of mathematics. It is triggered by the identification of topological questions in many areas of physics, ranging from quantum mechanics and quantum field theory over semiclassics to QCD and Condendsed Matter Physics. First, I tried to give an introduction to winding number statistics for newcomers who do not have any background, avoiding usage of expert jargon and of burying the key ideas under the adavanced terminology developed in mathematics and mathematical physics. Second, I reviewed results that my collaborators and I obtained in two recent works. We studied winding numbers and associated statistical quantities in a random matrix model. There are, of course, also other topological invariants of considerable interest in physics, most notably, the Chern numbers.

I presented our first, probably awkward, steps towards looking at universal behavior. In my opinion, the most fascinating challenge for the future is the further study of universality in statistical topology, more precisely, of both of its aspects, the experimental–empirical as well as the theoretical–mathematical one.

## Data Availability

No new data were created in this study.

## Abbreviations

The following abbreviations are used in this manuscript:

RMT	Random Matrix Theory
QCD	Quantum Chromodynamics
chGUE	chiral Gaussian Unitary Ensemble
chGSE	chiral Gaussian Symplectic Ensemble
$S_{N}$	permutation group of *N* objects
